# Clinical Review, and Hospital Reports

**Published:** 1843-01-01

**Authors:** 


### Clinical Review.

---

Clinical Midwifery, with the Histories of 400 Cases of Difficult Labour. By Robert Lee, M.D. F.R.S. &c. and Lecturer on Midwifery at St. George's Hospital. 12mo. pp. 224. Churchill, London, 1842.

This is a most instructive compendium, or catalogue *raisonnée*, of all the cases of difficult parturition, which have occurred in the practice of the author during the last fifteen years. There is no man in the profession more generally esteemed, or whose opinions are received with more confidence and respect by his brethren, than Dr. *Lee;* and well does he deserve, for most assiduously has he worked for, the eminence to which he has attained.

After distinguishing himself as a most able obstetrical physiologist (if we may so speak) he is now recognised as one of the best practical accoucheurs of the metropolis. We need say nothing more in recommendation of the present volume. It reminds us a good deal of the excellent practical observations of Dr. *Ramsbotham*, (senior,) and the valuable report of Dr. *Collins* of Dublin, both of which works we copiously reviewed some years ago in this Journal.

The importance, nay the necessity, of a competent knowledge of midwifery is now acknowledged by every examining body in the kingdom; even the College of Surgeons, which has been so tardy in permitting any change in their routine of examinations, has at length required it of their candidates.

If there is one branch of professional practice which should be thoroughly well understood by every medical man, before he commences on his own account, whether in the country, or abroad, or in either of the public services, we should say it is midwifery. A practical work, therefore, like the present, cannot fail to be truly useful; more especially as there is no little discrepancy of opinion between British and Continental accoucheurs on many of the leading and most important points of practice.

This seems strange, as nearly the same description of cases must occur in all countries alike—at least, in such as are in a state of civilization—and the same sorts of difficulties and of dangers must be met with everywhere. Yet the mode of treating these, recommended by obstetrical writers in France and indeed in most countries abroad, differs most widely from that pursued by all the leading authorities with us. Let us take a few instances alluded to by Dr. *Lee*.

The *forceps* is very rarely employed in this country, before the os uteri is soft and well dilated, or before the head of the child has descended so low into the pelvis that an ear can be felt. It is used solely with the view, as our author says, of supplying that power which the uterus does not possess at the time. Hence it is chiefly in cases of great exhaustion, or of hæmorrhage, or convulsions, or of other accidents where it is necessary to expedite the delivery of the child, that the British accoucheur has recourse to the use of the instrument. If the pelvis be considerably deformed, or if the soft parts be still rigid and unyielding, he prefers other means to effect the same end.

Now mark the difference in these respects on the part of French physicians :—

"The employment of the long forceps in cases of distorted pelvis has been recommended by Baudelocque, Boivin, Lachapelle, Capuron, Maygrier, Velpeau and Flammant, whose works contain ample instructions for its use, before the head of the child has entered the brim of the pelvis; and the last of these writers has expressed his belief, that the instrument is more frequently required while the head of the child remains above the superior aperture of the pelvis, than after it has descended into the cavity."

We need scarcely say how injudicious, nay how dangerous, such practice must necessarily be.

Take, again, the subject of *craniotomy*.

Dr. *Lee* thus clearly points out the circumstances which warrant and require the performance of this operation: "It is performed," says he, "by all British practitioners of reputation, whether the child be alive or dead, if the condition of the mother is such as to render delivery absolutely necessary, and the head of the child is beyond the reach of the forceps, or where, from distortion of the pelvis, or rigidity of the os uteri and vagina, it cannot be extracted if its volume is not reduced. This operation is performed from a conviction that, if neglected to be done at a sufficiently early period, the mother's life will be sacrificed, and the life of the mother is considered as much more important than that of the child."

The French condemn us for unnecessarily sacrificing the life of the child on many occasions! According to them, the operation of craniotomy should never be resorted to, until it has clearly been made out that the fœtus is dead, whatever may be the state of the mother at the time. *Baudelocque* expressly says, "nothing can excuse the conduct of the practitioner who would perforate the head of the child without previously knowing with certainty that it was not alive, a circumstance which can alone authorise us to employ the perforator and crotchet:" and *Velpeau*—whose authority, by-the-bye, we should not value much—goes even further than this, and maintains that, even when the child is dead, if the diameter of the pelvis is only 15 lines, or if the whole hand cannot be passed into the cavity of the uterus to turn the child, the Cæsarean operation is to be performed. Pity the poor French women who are entrusted to the tender mercies of such an accoucheur, say we. The practice of many of the leading accoucheurs in Germany is much alike. If the child be alive, and if there be no room for it to pass, the general rule seems to be to perform *hysterotomy*, or the Cæsarean section, rather than *craniotomy*.

Let us now for a moment consider the results—as well as we can judge from published reports; for, as Dr. *Lee* observes, not a few fatal cases on the Continent are never made known—of the former frightful operation.

*Michaelis* has collected the reports of 258 cases, of which 144 occurred in the last, and 110 in the present, century: of these cases 140 proved fatal. *Velpeau* tells us that the operation was performed 28 times between the years 1810 and 1820, and 62 times between 1821 and 1831—a statement which confirms what we have heard said, that the frequency of the operation on the Continent is, of late years, on the increase. Dr. *Churchill* says that it has been performed 316 times during the last ninety-one years, and that the mortality has been 52.8 per cent. for the mothers. (Is it not higher than this?)

Our readers need scarcely be told that the Cæsarean section has very rarely succeeded in this country. The cause is abundantly obvious; the unwillingness to have recourse to so terrible a resource, unless the life of the mother peremptorily requires it. "There is no eminent accoucheur now practising in London, who has been present at the performance of the operation upon the living body, or who would recommend it, if delivery could be effected by the perforator and crotchet"

Long may our countrymen continue to act on this principle. Where are the circumstances that can ever warrant the certain endangerment, nay often the more than probable sacrifice, of a mother's life for the chance—and, be it remembered, it is nothing more—of preserving that of her child? How few of the children that, "have been ripped from their mothers' belly," like the Thane of Cawdor, have been reared! and then think what a miserable end for a poor creature, after undergoing the sharpest pangs that flesh can know, to be subjected to a painful and bloody operation, not for her own, but for another's possible, advantage! Every principle of humanity and religion is opposed to such a practice; nay, even the cold dictates of mere science and physiology must condemn it. Why, then, it may be asked, should practitioners abroad have recourse to it with so little hesitation? The answer is simple, and will at once be surmised by those who are at all acquainted with medical practice on the Continent, and more especially in France. Patients, at least those among the poorer classes, seem to be regarded, not so much as fellow-creatures that have the same hopes and fears and the same feelings and destinies as ourselves, but rather as objects, so to speak, of natural history, which the learned doctor has to speculate and experiment upon.

The golden rule, not to do unto others what we should not wish done unto ourselves in similar circumstances, is too often forgotten by us all.

The last subject of discrepancy of opinion between British and Continental accoucheurs, that we shall notice at present, is the very important one of *inducing premature labour*, when the life of the mother is endangered by allowing pregnancy to go on to its full period.

"In numerous cases," we avail ourselves of Dr. *Lee's* own remarks, "it has been successfully employed in this country, and it is now ascertained that the operation is attended with little risk to the mother, and that nearly one half of the children are born alive and continue to live where it is performed after the seventh month. In cases of great distortion of the pelvis, the induction of premature labour at an early period of pregnancy, before the sixth month, is likewise known to be a safe operation, and to render craniotomy and the Cæsarean section wholly unnecessary."

In Germany and Holland, the operation has of late years been frequently resorted to, with very satisfactory results; and in these countries the practice is gradually rising in estimation. Not so in France. There is scarcely a single obstetrical writer who approves, or has performed, it. *Baudelocque* and *Dugès* condemn it, and Mad. *Lachapelle*, the celebrated "sage-femme" of Paris, tells us that she has never either employed it nor seen it employed. The Academy of Medicine held a long discussion, in 1827, on the propriety of the practice, and the learned body then decided that it was unjustifiable under any circumstances!* We have very little doubt but that this judgement will be reversed ere long, and strongly suspect, at the same time, that our enthusiastic confreres will be, one of these days, "going a-head" of their neighbours on this very subject: *nous verrons.*

Let us now rapidly glance at the several reports, or sets of cases, related by Dr. *Lee.*

*Report* I, gives the histories of 55 cases of difficult parturition, in which the forceps was applied. Many of these are very interesting, and deserve to be studied by the obstetrical practitioner, but we cannot afford space at present for the particulars of any. We observe, in one report, the following very candid admission.

"It would have been much better practice in this case, had I abandoned the attempt to deliver with the forceps, when the head could not be extracted by moderate traction. The unwillingness to resort to craniotomy, knowing that the child was alive, led me to commit what I believe to have been a practical error, and which would have been avoided, had the condition of the mother only been taken into consideration."

---

* If we mistake not, M. *Dubois* of Paris communicated, in the course of last year, to the Academy the reports of one or two cases in which he successfully induced premature labour, in consequence of extreme deformity of the pelvis.

Dr. *Lee* closes this chapter with a few practical remarks—it is to be regretted that they are not more ample—which our readers will thank us for giving. After mentioning that, 17 only of the children were born alive, and that, in several of the cases, the perineum and the vagina had been more or less injured from the injudicious employment of the forceps, he says: "In none did any benefit result from the instrument, before the greater part of the head of the child had passed through the brim of the pelvis, and the orifice of the uterus was fully dilated;" and again, "in protracted labours, when the head has made no sensible advance for hours, and becomes compressed, the scalp puffy and swollen, the vagina dry, hot, and tender, the discharges offensive, and when the bladder cannot be emptied without the catheter, it is dangerous to trust longer to the natural efforts. If, along with these symptoms, there is tenderness of the abdomen, fever, incoherence, restlessness, and exhaustion, delaying long to deliver is invariably followed by the most injurious consequences."

We need scarcely say that, when such symptoms as these are present, blood-letting, the use of the tartrate of antimony, &c. should be resorted to, before recourse be had to instrumental aid. Much mischief is often done in tedious labours by the injudicious exhibition of ergot of rye; a remedy which has been so lavishly and thoughtlessly used of late years.

*Report* II, describes 65 cases of "difficult labour from distortion of the pelvis, swelling of the soft parts, convulsions, hydrocephalus in the fœtus, and other causes, in which delivery was effected by craniotomy."

Many of these cases very forcibly inculcate the importance of avoiding too long delay in effecting the delivery, and the danger that is apt to follow from neglect of this rule.*

A difficult and protracted labour is, in many respects, a good deal like a case of strangulated hernia: the danger is more in the operation for relief being put off too long, than being performed too early. Dr. *Lee* very feelingly remarks of one case, where the patient died in twelve hours after the extraction of the child with the crochet; "the result of this case would probably have been very different, had we proceeded to deliver twenty-four hours sooner, (the symptoms

---

* As a curious and not uninstructive case, the following will be read with interest:—

"A dwarf from the Mauritius, Santiago de los Santos, married an Englishwoman at Birmingham, whose height was three feet and three inches. She became pregnant, went to the full period, and was in labour at Chelsea on the 14th of April, 1835, under the care of Mr. Bowden. Dr. H. Davies was consulted, and finding the pelvis greatly distorted, he opened the head of the fœtus on the afternoon of the 15th of April, and removed with the crotchet a part of the cranium. At nine P. M. we proceeded together to complete the delivery with the crotchet, the outlet and brim of the pelvis being so contracted that all the varieties of craniotomy-forceps were perfectly useless, as they could not be applied. The operation lasted nearly five hours, and the head of the fœtus could not be drawn through the brim of the pelvis, until the bones of the base of the skull were all torn to pieces with the crotchet; the point of which was generally passed up on the outside of the head. An arm was next drawn down, and the thorax torn open and all the viscera extracted. So great was the degree of distortion, that the pelvis of the child could not be drawn through the brim of the mother's pelvis till after long-continued efforts with the crotchet. We were both thoroughly exhausted before the delivery was accomplished, and it seemed at first impossible by any means to extract the child without producing fatal contusion or laceration of the uterus and vagina. On the 12th of May, the patient was walking about, and perfectly well."

had not been alarming) and I can never think of it without regret;" and again, after recording five fatal cases where it had been necessary to perforate the head in consequence of congenital hydrocephalus, he does not hesitate to give it, as his opinion, that, had the cause of the difficulty been ascertained sufficiently early in these cases, and the operation of craniotomy been performed, it is impossible to doubt that some, if not all, of them would have ended favourably. The chapter closes with the following practical observations:—

" Rupture of the uterus took place in three before perforation, and the inflammation and sloughing of the uterus, vagina, and bladder, which proved fatal to eight others, were chiefly or solely produced by the long-continued violent pressure on the soft parts by the head of the child, before it was opened and extracted. In those who recovered with vesico-vaginal fistulæ, or contractions of the vagina from cicatrices, the unfortunate occurrences arose from craniotomy being too long delayed. After examining all the details of these cases, I feel satisfied that in none was the interference premature, and that in several, had the delivery been sooner effected, the fatal consequences which ensued would have been wholly prevented."

The whole of this chapter reminds us strongly of Dr. *Collins*' excellent work; and it is gratifying to find that two such experienced men agree in the practice they recommend.

The *Third Report* treats of the " induction of premature labour in cases of distorted pelvis, cancer of the uterus, uterine and ovarian tumors, organic and nervous diseases of the heart, dropsy of the amnion, obstinate vomiting, hæmorrhage from the bowels, and chorea, during pregnancy." Not fewer than 58 cases are related in this chapter, which, on the whole, is certainly the most interesting and instructive in the book. Our readers must take our word for this, until they consult it for themselves, as we can afford room only for a very few extracts.

" The greater number of the best practical writers on midwifery in this country have considered the induction of premature labour applicable only to cases of slighter distortion, and have considered it improper in first pregnancies, and before seven complete months of utero-gestation have elapsed. Little has been said by them respecting the safety and utility of the operation in cases of great distortion, to obviate the danger to the mother of fatal contusion or laceration of the uterus and vagina, which are always to be dreaded when much force is required after perforation to extract the head of the child."*

In one woman, whose pelvis was considerably deformed, Dr. *Lee* induced premature labour in no fewer than ten successive pregnancies: her first labour, at the full time, had been a very severe one, and was completed only after extreme difficulty and danger with the perforator and crotchet. The plan, that our author usually follows, is to perforate the membranes: he appears to have little or no confidence in the *secale cornutum*. On this point, Dr. ***Ramsbotham***, certainly a good authority, differs from him.†

---

* Dr. *Lee* subsequently remarks that, " in no case of distortion, however great, can it be necessary to induce premature labour before the end of the fifth month of pregnancy, when the fœtus is so small and soft that it can be easily extracted. The length of the cervix uteri before this period must render it both dangerous and difficult."

† At a subsequent page of his book, Dr. *Lee* observes, that he has recently been informed that premature labour may be induced by the same means, (the introduction of a large soft dry sponge covered with lard into the vagina, and firmly pressed up against the os uteri—by far the best remedy, by the bye, against profuse hæmorrhage in many cases of threatened abortion) without forcing the sponge into the uterine orifice. " I have had no opportunity of trying this

The case of a Mrs. *Jarvis* is especially interesting: after giving birth to three living children at the full period, she became affected with softening of the bones and concomitant distortion of the pelvis. She again became pregnant and went to her full time; but now it was only by breaking the head of the child to pieces that it could be drawn down with the crotchet into the pelvis. On her three successive pregnancies, premature labour was brought on, and every thing did well; but the next occasion was more unfortunate. Unusual difficulty was experienced in puncturing the membranes, and a good deal of time was lost in trying the effects of the secale cornutum. At length the liquor amnii was discharged, and labour-pains commenced; the extraction of the child was effected with the most extreme difficulty by means of the crotchet and perforator. At one time Dr. *Lee* thought that it would be necessary to have recourse to the Cæsarean operation, to prevent the woman dying undelivered. The pelvis was found on dissection to be exceedingly deformed; it is now in the museum of St. George's Hospital. With the candour of an honorable man, Dr. *Lee* admits that it would have been much better, had premature labour been induced in this case at an earlier period.

Some of the late cases in this chapter are exceedingly interesting. There is one in which there was such violent irregular action of the heart, aorta, and carotid arteries, that several eminent physicians, who were consulted, believed that there was aneurism of the arch of the aorta. Every remedy that was tried proved utterly inefficacious, and it was determined to induce premature labour as affording the only likely means of relief. The attempt was made, but failed. This was in the sixth month of pregnancy. Notwithstanding the most unfavourable symptoms, the lady went on to her full period, had a natural labour, and was ultimately entirely relieved of all the cardiac symptoms.

There are several cases of dropsy of the amnion recorded; in some it was co-existent with ascites—a complication that will necessarily obscure the diagnosis very much. But even when the disease is simple, and when there is no abdominal effusion present, the fluctuation of the water may often be perceived through the abdominal parietes, if the uterine accumulation be very great. In all suspected cases, an accurate examination must be made per vaginam. The treatment of the disease is abundantly simple; all that is necessary to be done is to puncture the membranes and evacuate the superabundant liquor. Dropsy of the amnion is viewed by our author as one of the numerous diseases of the fœtus and its appendages, which occur quite independently of the health of the mother. Hence the child in such cases, although born alive, seldom survives long.

Unappeasable vomiting during the early months of pregnancy sometimes demands the puncture of the membranes. In case 174, the operation was done with speedy relief to the symptoms, and the lady went to the full period, and was at length safely delivered of a living child. This however is certainly not a frequent occurrence; for abortion almost always follows its performance. And let it not be imagined that the operation is quite exempt of danger. Dr. *Lee* mentions, in a report of a case where Dr. *Merriman* also was consulted, that he, (Dr. M.), while he assented to the propriety of inducing abortion if every other means failed, urged the greatest caution, in consequence of a case which occurred some years ago in the practice of a celebrated accoucheur, and ended fatally after the performance of the operation, and for which he unjustly incurred much odium. As a matter of course, no prudent practitioner will ever act on his own responsibility alone in such circumstances; indeed, this rule holds good in almost

---

method, but if it should always be successful in bringing on labour, there can be little doubt that it will possess great advantages over all the other means which have hitherto been employed for this purpose."

every difficult and complicated midwifery case: let a second, and even a third, opinion be if possible taken, before recourse be had to remedies, the use of which is not exempt from danger.

The *Fourth Report* is taken up with the narration of "difficult labours from presentations of the superior extremities, nates, and funis." The great lesson to be derived from this chapter is the importance of an early detection of the presenting part, so that, if turning be necessary, it may be had recourse to before the membranes have burst, and the water of the amnion escaped.

"The histories of sixty cases of arm-presentation are contained in this report. In a large proportion of these, the operation of turning was undertaken in the most unfavourable circumstances, both for the mothers and their children, after the liquor amnii had entirely escaped, and the uterus had not only been contracting for many hours around the child, but repeated unsuccessful efforts had been made to deliver. Seven women died from rupture of the uterus, and three from inflammation of the uterus. Laceration and inflammation of the uterus are, therefore, the consequences to be dreaded after turning."

The *Fifth Report* details 36 cases of hæmorrhage before delivery from the presentation of the placenta at the cervix, or over the orifice, of the uterus. We need not say how alarming this accident is on all occasions, and above all, if the flooding occurs while the os uteri is only slightly dilated, or if there be any co-existing malformation of the pelvis. Perhaps there is not a case within the whole range of medical or surgical practice that requires more moral courage and professional skill, on the part of the attendant, than uterine hæmorrhage from placental presentation. There is often no time for consultation; the physician has to act for himself; a fellow-creature's life is at stake; and it may depend upon him whether it be saved or not.

Dr. *Lee* points out the advantage of puncturing or rupturing the membranes in many cases of partial presentation of the placenta; or of alarming hæmorrhage from any cause, before delivery—a practice first strongly recommended, we believe, by that experienced accoucheur, Dr. *Merriman*. This alone will very often be followed by an arrest of the hæmorrhage; and, in not a few cases, the delivery will be safely completed by the natural efforts of the uterus, and without the operation of turning. The use of the plug in such cases, we need scarcely say, is utterly useless; unless indeed to gain time for the dilatation of a rigid os uteri to take place. The employment of the ergot is often positively hurtful.

We observe that Dr. *Lee* disapproves of the practice of introducing the hand into the cavity of the uterus and pressing on or tapping its inner surface, with the view of exciting the organ to contract, in cases of alarming hæmorrhage, after the expulsion of the placenta. He says that "it is not only ineffectual for the purpose in the worst cases of this kind of flooding, but that it is attended with mischievous consequences after the hæmorrhage has been suppressed." He trusts to the use of constant and powerful pressure over the uterus, the application of cold water to the external parts, and the exhibition of powerful stimulants, especially wine and brandy: smooth pieces of ice may also be introduced into the vagina.

The remaining reports of cases of retention of the placenta, and of labours complicated with puerperal convulsions need not detain us. Many of the examples are certainly very interesting; but we miss much some practical remarks on the subjects alluded to, and which might have been introduced most opportunely at the end of each chapter, in the way of comment on the reported cases, and general rules to guide the practitioner. We cordially invite the author to make the second edition of his work more complete, by introducing here and there his own opinions more at large on the many grave questions which come under notice; the sentiments of so experienced and talented a man as Dr. *Lee*

are sure to have high authority among his professional brethren. What are the results of his observations on obstetrical auscultation? There is scarcely any allusion to the subject in the present work.

---

GUY'S HOSPITAL REPORTS, No. XV. October 1842. Edited by GEORGE H. BARLOW, M.A. and M.D. Trin. Coll. Cam., and JAMES P. BABINGTON, M.A. Trin. Coll. Cam. 1841. Highley, London.

THE Contents of the present Number are as follows:—

On Pneumonia; by H. M. Hughes, M.D.—Cases of Hæmorrhage, occurring after Delivery, and complicated with Disease of the Spleen and Kidneys; by John C. W. Lever, M.D. F.S.S.—Note on the Microscopic Globules found in Urine; by Golding Bird, A.M. MD. F.L.S.—Case of Poisoning by Arsenic; by John Hilton, F.R.S. With the Chemical Examination of the Contents of the Stomach, Blood, &c.; by Alfred S. Taylor.—Case of Fatal Pleuritis, apparently the effect of the presence in the Right Pleura of a Piece of Ivory, consisting of Four Artificial Teeth, which had been swallowed thirteen years before; by W. G. Carpenter.—Observations upon Inflammation of the Aqueous Membrane of the Eye. Read before the Physical Society of Guy's Hospital, April 2, 1842; by Joseph R. Bedford.—Observations on the Diseases of the Orifice and Valves of the Aorta; by Norman Chevers, M.D.—Case of Contracted Aorta; communicated by William Muriel, Esq. of Wickham-Market, Suffolk. Two Cases of Disease of the Larynx, requiring Laryngotomy; with Observations; by John Hilton, F.R.S.—On the Operation for Cataract; by John Morgan, F.L.S.—Observations on certain Diseases originating in Early Youth, illustrated by Cases of Defective Expansion of the Lungs. (Memoir the Second.) By George H. Barlow, M.A. and M.D.—Sequel of the Case of Lochland Shiel, who was operated on for Exostosis of the Bones of the Face, on the 1st of August, 1835.

We shall give some account of most of the preceding Papers.

### I. ON PNEUMONIA. By H. M. HUGHES, M.D.

The chief object of Dr. Hughes, is to contrast pneumonia with phthisis, as regards its location, and exhibit the diagnostic importance of the latter. Dr. Hughes has been struck with the difference between the recorded experience of French authors and his own, and insists on the necessity of comparing the laws of disease in one country and under one set of circumstances, with those of the same disease in another country and other circumstances. Dr. Hughes informs us:—

"I have prepared two tables. One contains cases of pneumonia, recognised as such during life. These have been generally obtained from the books of the clinical wards, and those of the Clinical Society. This table embraces the names, age, and sex of the patient; the complexion of the individual, when stated; the lung, and the part or parts principally or primarily affected; the form of the complaint; the most important complications; the principal treatment; and the results. The other consists of an account of those cases, inspected in the hospital, in which distinct evidence of the previous existence of inflammation of the lungs was discovered after death. In some of them, pneumonia was the principal or fatal disease, but in much the larger proportion it was only a complication of other affections of a more chronic character. As these cases have been almost always derived from the inspection books, in which it is not general to introduce a detailed history of the previous disease, the distinction between these orders of cases was not usually well marked. I have, therefore, thought it better to include the whole in one simple category of cases, in which pneumonia was merely discovered after death, and to exclude those which find a place in the other table. It may be, however, well to repeat, that, almost all the cases in this table have been instances in which pneumonia supervened upon or co-existed with other diseases. This table contains 145 cases; and embraces the name, age, and sex of the individual; the lung and part diseased; the form of the complaint and the principal disease or diseases co-existent with that which is the subject of inquiry. In collecting these materials, I have carefully examined thirty manuscript quarto volumes of inspections preserved in the Museum of the hospital."

The instances of pneumonia reported to have occurred among these cases are nearly 150. But the inspections were not sufficiently complete to permit us to place absolute dependence upon these returns. Prior to furnishing an analysis of the tables, Dr. Hughes makes some practical observations on pneumonia, as it has occurred in his own experience.

*Morbid Anatomy.*—Dr. Hughes believes, with Dr. Hodgkin, and, we think, with truth, that there are two kinds of grey hepatization or grey softening. "In one of these states, the grey succeeds to red hepatization: the inflammatory deposit softens down, and induces suppuration in the surrounding tissues. The other is the immediate result of inflammation in a person of feeble power or bad constitution; it is not preceded by red hepatization; but derives its peculiar soft and friable texture, and its grey or dirty white colour, from the nonplastic or unorganizable nature of the material originally deposited in the inflamed lung. The one is *usually* found to co-exist with plastic deposits; the other with sero-purulent effusions in the serous membranes."

He conceives, with Drs. Addison and Hodgkin, that the primary seat of pneumonia is the membrane lining the pulmonary vesicles, and adduces several arguments in support of his opinion. But into these we will not enter.

*Is Chronic Pneumonia rare?*—Dr. Hughes comments on the idea entertained by French pathologists of the extreme rarity of chronic pneumonia. "Their opinions," he observes, "on this subject are probably somewhat exaggerated in the following question proposed by Laennec—'Is there really such a disease as chronic pneumonia?'—'If,' in the words of the same celebrated author, 'we may term those cases *chronic*, in which the peripneumony, although originally acute, has been checked in its progress by blood-letting and other antiphlogistic means, but in which these antiphlogistic means have been insufficient to procure speedy resolution, or even to prevent relapses;' then I have no hesitation in saying, that, in my own experience, chronic pneumonia is not *extremely rare.* If to these cases are added those, in which masses, varying in size from a pin's head to a walnut or even a pullet's egg, of various shades of grey, red and purple, firm and dry, sometimes to such a degree as to creak under the scalpel, are found distributed in various parts of the lungs without any traces of tubercles being discovered, then I believe that, at least in London, chronic pneumonia is not even *rare.* But if with these may be included the cases in which dry, grey, firm, almost semi-cartilaginous consolidations are witnessed around old tubercular cavities, though these consolidations are themselves quite free from tubercular deposit—and those in which the same condition of the pulmonary tissue is found to be interspersed among, but clearly distinguishable by its hardness and dryness from masses affected with tubercular infiltration, or the more ordinary form of tubercle, then I believe that chronic pneumonia, far from being a *rare*, is a very *common* complaint."

*General Symptoms.*—Dr. Hughes gives a good general sketch of these, and then dwells on some of the more prominent. Of course, the sputa claim his attention, and he describes them with accuracy. But he admits that "pneumonia is far from being universally accompanied with sputa presenting these characters: not unfrequently, they are altogether absent: more commonly, it is imperfectly mixed with the white, frothy, and moderately tenacious expectoration of acute bronchitis. In not a few cases, there is scarcely any expectoration at all, and in some there is none. The more extensive the bronchitic complication, the more copious and frothy are the sputa; and, as far as I have observed, the lower the disease—that is, the more it approaches the typhoid form of the complaint—they are the more scanty and dark-coloured. In my own experience, also, it has rarely happened that lobular pneumonia has been attended with the '*crachats rouillés*' of the French authors. The expectoration in such cases has rather presented the appearances of acute capillary bronchitis."

The pungent, stinging heat of skin, so insisted on by Dr. Addison, is also alluded to by Dr. Hughes, who insists on the frequency of its occurrence while he admits its not uncommon absence.

The trifling cough, so different from the paroxysms of bronchitis, the teasing cough of nervous disorders, or the constant hacking of phthisis, is touched on. Dr. Hughes adds:—

"Another circumstance respecting this symptom may be worthy of notice. Although the patient has asserted that he had no cough—and though none he has had, or has appeared to have, while breathing gently—yet I have never known an individual suffering from this complaint who could take a deep inspiration—*i. e.* not merely, after shortly inspiring, raise the shoulders by muscular effort, without at the same time elevating the ribs, as these sufferers are extremely apt to do when told to 'heave a sigh,' but really attempt to fill his chest with air—without the effort being followed by the slight double hacking to which I have referred. It appears, that so long as, under ordinary respiration, the inflamed part remains quiet, little or no cough may be induced by it; but that when, by forced inspiration, there is a tendency to dilate the diseased air-cells by the inhaled air, the cough, or rather hacking, is the almost necessary result. These observations, of course, only apply to those cases in which there is little or no complication with bronchitis. If this disease is also present, the cough will be frequent, and of a different character."

Dr. Hughes's version of the *Pulse* of pneumonia is—*frequent, contracted,* and *incompressible:* at the same time it is a pulse which is remarkably affected by the abstraction of blood. Under the lancet, its character is often completely changed; and even while the blood is flowing, it expands sensibly under the finger, and becomes less frequent, as well as more soft and compressible.

*Physical Signs.*—Dr. Hughes gives a succinct account of these. He alludes to the opinion of Dr. Stokes, that there is a stage preceding the usually admitted first one of pneumonia, characterised by "an intense puerility of respiration in the affected part," and marked by the "lung being drier than natural, with intense arterial injection, and no effusion of blood into the cells." This earliest stage, Dr. Hughes thinks is very likely, but he has never witnessed it.

Dr. Hughes is very precise in his notions of what is true crepitating rattle. His observations on this point deserve attention.

"The question is not unfrequently asked, Is the crepitating rattle pathognomonic of the first (Dr. Stokes's second stage) of pneumonia? To this question I reply, by asking, What is intended by crepitating rattle? If that which I have frequently heard so called by young auscultators—viz. a small but unequal crackling or crepitation, accompanying part of the exspiration as well as a great portion of the act of inspiration—is thereby intended, then the crepitating rattle is certainly not pathognomonic of that stage of the disease. If, again, a finer and more equal, but a soft and moist crepitation, heard, principally at least, at the end of the inspiratory and beginning of exspiratory effort, is the sound concerning which the inquiry is made, then the answer must be, that crepitation is not pathognomonic of pneumonia at all. The one is the muco-crepitating rattle of capillary bronchitis, which, it is true, often accompanies the disease concerning which the inquiry is made, but is not the disease itself: the other is the sub-crepitating rattle of œdema and pulmonary apoplexy, which are often perfectly unconnected with it. Yet there is a crepitating rattle quite different from either of the preceding; and so peculiar, that it is only necessary once to have heard it distinctly, to be able to recognise it ever after; which, so far as my observation extends, is *perfectly pathognomonic.* This rattle, in its pure form, is, I believe, not known, or correctly appreciated, by a very large proportion of those who are in the habit of practising auscultation. On this account I shall endeavour somewhat minutely and, as far as I am able, accurately to describe it. The sound has, by different writers, been compared to the crepitation of salt in the fire, or upon a piece of red-hot iron—to the crumpling of a dry membrane—to the noise produced by the squeezing of gauze paper—or by the separation of two sticky surfaces; but the most correct representation of it, by far, is, in my opinion, that which was, I believe, first noticed by Dr. J. C. Williams, who compares it to the delicate crackling sound caused by an individual rubbing hardly between the finger and thumb a lock of his own hair, close to his ear. This appears to me very exact. Still, the sound is not recognised. I will therefore add another illustration, though I consider it, in almost every respect, inferior to that last mentioned. When varnish which contains a great number of excessively small globules of air is spread upon a plain surface and allowed to become nearly dry, and these globules are simultaneously burst by the pressure of a soft substance, such is the crepitating rattle of the first stage of pneumonia. It appears to the ear like a multitude of exceeding minute bubbles, all of the same size, by the same effort, and nearly at the same time, bursting in thin but very tenacious fluid; or as if the parietes of the air-cells themselves were stuck together through the medium of the same fluid, and the ingress of the air caused their separation. The *rattle is very fine, very dry, very equal; of short duration; and heard only in puffs, at the end of the inspiration.* When such a rattle is heard, I believe acute pneumonia may be certainly predicated.

"I am aware, that on this, and on some other subjects connected with the disease under consideration, I differ from writers who are justly regarded as authorities in complaints of the chest: but I give the result of my own repeated observation, independently of any theory, when I say that the *pure crepitating rattle of advancing pneumonia is first heard,* and I believe only heard at the end of the inspiration; that in this particular, as well as in the much greater fineness, dryness, and equality of the sound, it differs from the muco-crepitation of capillary bronchitis; and that it is to be distinguished from the subcrepitation of œdema and pulmonary apoplexy, by its comparative dryness and acuteness, and by its not accompanying the expiration.

"By the preceding observations, I by no means intend to imply that the first stage of pneumonia is always accompanied with a rattle having these characters; nor that the muco-crepitation, combined with other general symptoms and local signs, affords not often a perfectly satisfactory evidence of the existence of the complaint; but merely, that the crepitating rattle, to be pathognomonic, must possess such characters: and that when it does possess them, in my experience at least, it is thus pathognomonic."

Certainly the comparisons of auscultators are queer ones. They not unfrequently illustrate the ignotum by the ignotius, and these fine shades of crepitation, and equally fine differences of opinion about them, must sorely vex the young gentlemen and old ones who brandish stethoscopes with an imposing air, and listen to chests with a puzzled look. But *allons cultiver notre jardin.*

Dr. Hughes thinks that pneumonia is *not always* accompanied with crepitating rattle; and that crepitation does *not always* re-appear on the commencing resolution of the disease. He does *not* "recollect ever to have known the dulness on percussion, dependent upon active and sthenic pneumonia, to disappear, and bronchial respiration to be exchanged for the vesicular murmur, in the course of twenty-four hours, as Dr. Stokes states that he has himself witnessed;" *nor* has it fallen to his lot "to have observed any of those cases, in which a tympanitic resonance on percussion, the result, as has been supposed by some, of the sudden secretion and absorption of air in and from the pleura, has rapidly appeared and disappeared during the progress of pneumonia." Of the *secretion* of air into the pleura in such cases he seems, not unnaturally in our opinion, sceptical. He asks and answers two other questions, whether satisfactorily, or not, we will not take upon us to determine. At the same time, we cannot but express our disbelief generally in the *practical* value of refined distinctions of sounds which trench so closely on each other that words are incapable of defining them. It has not happened to us to meet, in real life, with the successful application of these minute diagnostics, nay, by a singular fatality, the greatest blunders have been often made by those who have attempted them. For the sake of those, however, who think they can attain to such precision, we subjoin these questions and answers.

"Is the crepitating rattle of receding distinguishable from this of advancing pneumonia? and can the crepitation of the third stage, or of suppuration, be distinguished from either or both of the preceding?

"In answer to the first of these questions, I would say, that if a patient, whose disease was just 'upon the turn,' or had just began to recede, was to be examined for the first time, I believe it would be impossible to decide, from the rattle alone, whether the complaint was advancing or retrograding; as at this time, according to my observation, the rattles are in every respect identical. Even in this case, however, the history of the disorder would probably afford some assistance; and dulness on percussion and bronchophony would contribute to indicate the existing condition of the diseased lung. But as the process of resolution goes on, the rattle becomes looser, moister, and softer: it also occupies a constantly increasing portion of the inspiration, together with the beginning of the exasperation. Under these circumstances, as I have before hinted, I believe it generally to be clearly distinguishable from that of the advancing malady.

"To the second question I would, notwithstanding the doubts expressed by M. Grisolle, answer generally in the affirmative. At the same time, I must acknowledge, as in the former instance, that if an opinion must necessarily be formed from the rattle alone in a patient who had not been seen before, there would be sufficient reason to hesitate before that opinion was delivered. Still, I believe its characters to be sufficiently marked, although it is difficult to give a correct representation of them in words. It occupies, even on its first appearance, the commencement of the exasperation as well as the end of the inspiration. It is larger and more irregular in the size of the bubbles, and, consequently in the quality of the sound, than either of those which I have previously described. In this respect it approaches most nearly to the muco-crepitation of capillary bronchitis than the two preceding rattles: but it is more *shrill* and *resonant* than any of them: it appears, indeed, to be what I presume it really is: viz. a muco-crepitating rattle, rendered more resonant and shrill by the superior conducting power of the solidified lung with which it is associated, in fact, a *bronchophony of muco-crepitation.*

"It resembles, more nearly than any other morbid sound with which I am acquainted, the muco-crepitation of softening tubercles, in those cases of phthisis in which there exists great consolidation of the surrounding lung, and in which, therefore, the same physical condition of the part may be instrumental in its production. If, then, to these characters of the rattle be added the continued dulness on percussion, the bronchophony, and some remains of tubular respiration, and especially if with these are conjoined the increase, and altered qualities of the expectoration, no doubt can, I think, be legitimately entertained as to the state of the lung, even though there be no 'coincident exasperation of the general symptoms,' which M. Grisolle appears to think a *sine qua non*; but which, in my experience, is certainly not a *necessary* accompaniment of the alteration."

Dr. Hughes observes, that when pneumonia affects the apex of the lung, pectoriloquism, and, if bronchitis be present, gurgling, are sometimes so distinct as to deceive completely.

*Treatment.*—Dr. Hughes scouts the idea of treating all cases of pneumonia in the same way. This of course applies to the treatment of every disease as well as to that of pneumonia. Age, habit of body, causes, and circumstances, must each and all influence treatment. He points out also the danger of trusting to comparative tables of the results of treatment in acute diseases. There are too many circumstances to be taken into the account in each individual case. With this proviso Dr. Hughes thus sets forth the plan that has been for many years resorted to in Guy's Hospital. The method, then, "has been to bleed the patient to approaching syncope; and to administer a pill, containing half a grain of opium and a quarter of a grain of tartarized antimony, with one or two grains of calomel, every three, four, or six hours, according to the severity of the symptoms. With this has been usually combined a saline mixture containing twenty or thirty minims of antimonial wine. If, in the course of a few hours, or on the next day, the general symptoms have been unsubdued, or, after a temporary mitigation, have returned in their former severity, venesection has been repeated. It has sometimes, though not often, been necessary that the operation should be again and again performed. Triple venesections have been uncommon, and a fourth very rare. If the general symptoms, on the contrary, have been reduced, though the local affection has continued severe—or if the power of the patient has been materially diminished by venesection—the abstraction of blood by cupping has been ordered, to the amount of from six to twelve ounces. As a decrease of the disease has been evidenced by a diminution of the general distress or a mitigation of local suffering, the medicines have been less frequently repeated. They have been discontinued altogether when a more notable or persistent change for the better has been apparent, even though the mercury has not produced its specific effect upon the mouth. If the system has evidently become affected thereby, but the complaint has been still active, it has usually been discontinued, or repeated only in small and comparatively unfrequent doses. Blisters also have been applied with good effect, in the latter stages of the disorder."

Dr. Hughes does not doubt, nor do we, the utility of blisters, although it has been disputed.

Dr. Hughes does not seem to have tried the "contra-stimulant" administration of antimony. He has been too well satisfied with the results of the ordinary treatment, to venture to change it. But he observes:—

"I must, however, confess, that the results of the treatment of pneumonia in Guy's Hospital—if all cases, however far advanced, and with whatever complications, are taken into the account—are not to be compared with what are stated to have been the really 'triumphant' effects of antimony in this severe malady. Thus Laennec says, that of sixty-two cases treated by antimony, only six died; and that of these six, two were moribund on their admission—two were old men of seventy, of whom one died from cerebral congestion—the fifth laboured under chronic pleurisy—and the sixth under disease of the heart. Others are reported to have lost only one in thirty, and one in forty cases. My own experience in, and my opportunities of observing the effects of the remedy in acute pneumonia, have, as I have already hinted, been limited. In one respect, however, my observation is entirely opposed to that of Laennec, who states that he has never known renewed attacks of the disease to occur when antimony had effected some, though slight, amelioration. I well recollect, however, a case of which I possess notes, treated by bleeding and tartar-emetic, in the Infirmary of Edinburgh, while I was a pupil there, in which the patient, after two venesections, the application of leeches, and the continued use of antimony, was so very much relieved as to be considered almost convalescent. The antimonial solution was however continued. But when the disease had for four days appeared to be rapidly decreasing and the patient was in every respect improving, the attack was renewed; and it was necessary again to bleed him to the amount of ten ounces, after which he rapidly recovered. The same has certainly happened in several cases that have fallen under my notice. In some instances, also, when antimony has been at first employed with benefit but relapses have taken place, it has been, or has appeared necessary for the cure, to administer calomel and opium in combination with it."

He relates a case illustrative of the good effects of mercurialization. Repeated relapses occurred, until the mouth became affected. After some further remarks and another case, Dr. Hughes thus sums up his opinions on the use of antimony, opinions with which we must say that we are disposed to concur:—"*that* antimony is a very active remedy in the treatment of pneumonia; that it is more particularly indicated in slight and recent cases—those complicated with bronchitis—those in which venesection cannot be borne or repeated—and those in which mercury cannot be safely employed: that the cases treated by it are often more rapidly relieved than by any other means, but that they are also more than ordinarily liable to relapses: that when consolidation has obviously occurred, it should not be trusted alone, but should always be given, together with mercury: that, in fact, though often very striking and rapid in its effects in the earlier stages of the complaint, it cannot be administered as a certain remedy at any time, but more particularly when there exists extensive solidification of the lung, unless in combination with calomel and opium."

*Statistics.*—The first Table furnished by Dr. Hughes refers to one hundred and one cases recognised as primary pneumonia.

1. *Side affected.*

| | |
|---|---|
| The right lung was alone diseased in . . . . | 52 cases |
| The left lung was alone diseased in . . . . . . | 29 .. |
| Both lungs were diseased in . . . . . . . . . | 19 .. |
| Side not mentioned in . . . . . . . . . . . | 1 .. |
| | 101 |

2. *Parts affected.*

| | | | |
|---|---|---|---|
| The base* alone of the . | Right lung | 36 | 62 .. |
| | Left lung | 16 | |
| | Both lungs | 12 | |
| The whole of the . . . | Right lung | 4 | 12 .. |
| | Left lung | 6 | |
| | Both lungs | 2 | |
| The posterior alone of the | Right lung | 3 | 8 .. |
| | Left lung | 4 | |
| | Both lungs | 1 | |
| The apex alone of the . . | Right lung | 4 | 5 .. |
| | Left lung | 1 | |

* "It is not hereby intended that the disease was in any case *strictly confined* to the part mentioned, and that it did not encroach on those adjoining; but simply, that in the notes of the cases this is mentioned as the diseased part indicated by the physical signs."

| | | | |
|---|---|---|---|
| Centre alone of the . . | { Right lung . 2 / Left lung . 1 } | 3 | .. |
| The parts were not mentioned in . . . . . . . | | 2 | .. |
| Various parts in one or both lungs in . . . . | | 9 | .. |
| | | 101 | |

3. *Both Lungs inflamed.*

| | | |
|---|---|---|
| In the bases of both, in . . . . . . . . . . . | 12 | cases. |
| Generally throughout both . . . . . . . . . | 2 | .. |
| In the posterior of both . . . . . . . . . . | 1 | .. |
| In all of one, and the apex of the other . . . . | 2 | .. |
| In the apex of one, and the centre of the other . | 1 | .. |
| In the posterior of one, and base of the other . . | 1 | .. |
| | 19 | |

4. *Sex.*—76 cases were in males—25 in females. The latter, of course, are least exposed.

5. *Complexion.*—Seems to make little difference, for, of 42 named instances, 23 were in light complexions, 19 in dark.

6. *Age.*

| | | |
|---|---|---|
| Below the age of 20, in . . . . . . . | 24 | cases. |
| Above 20, and below 30 . . . . . . | 38 | .. |
| Above 30, and below 40 . . . . . . | 19 | .. |
| Above 40, and below 50 . . . . . . | 8 | .. |
| Above 50 . . . . . . . . . . . . | 12 | .. |
| | 101 | |

7. *Form.*

| | | |
|---|---|---|
| Disease in an acute or sub-acute form, in | 92 | cases. |
| In a chronic form . . . . . . . . . | 8 | .. |
| With acute gangrenous abscess . . . . | 1 | .. |
| | 101 | |

8. *Complications.*

In every case but one, in which any considerable portion of the surface of the lungs has been inflamed, pleurisy has co-existed.

| | | |
|---|---|---|
| Bronchitis was ascertained to co-exist in | 22 | cases. |
| Phthisis . . . . . . . . . . . . . | 5 | .. |
| Influenza . . . . . . . . . . . . . | 4 | .. |
| Continued fever . . . . . . . . . | 3 | .. |
| Pericarditis . . . . . . . . . . . | 3 | .. |
| Erysipelas, disease of the aorta, delirium tremens, epilepsy, hæmaturia, aneurism, empyema, rheumatism, renal disease, simple catarrh, pertussis, and fracture—of each, one . . . . . . . | 12 | .. |
| Cases uncomplicated, or complicated with pleurisy alone . . . . . . . . . . | 52 | .. |
| | 101 | |

*General Results.*

| | |
|---|---|
| There were cured . . . . . . . . . | 70 |
| Died . . . . . . . . . . . . . | 24 |
| Were relieved, or were not reported . . | 7 |
| | 101 |

*Results as connected with Treatment.*

Of the cases that were considered fit for the general plan of treatment before-mentioned, there were 47. In these there were 41 recoveries, and 6 deaths. Among these latter, were two cases of phthisis and one of delirium tremens.

Of the cases treated without mercury (slight) seven were successful, two fatal. One of these was a case of pericarditis—one of aneurysm. Two were unsatisfactorily treated with antimony, recovered with calomel and opium.

There were *not bled* 37. Twenty recovered—13 died—the fate of four is unknown.

*Results in relation to Location.*

"The cases in which both lungs have been diseased were nineteen;—of whom twelve recovered, and seven died.

When the whole of one lung was diseased, of which there were ten—four of which were on the right side and six on the left—five recovered, two died, and in three the result is not mentioned.

Of the five cases in which the apex was the part alone affected, and of which four occurred in males, and one only in a female, four recovered; and one, the female, died. The respective ages of these five patients were, 31, 32, 35, 45, and 50. They thus tend to confirm the opinion of M. Louis—if that opinion is reduced to a simple statement—that pneumonia of the apex generally occurs at a more advanced age than the average age of those affected with this complaint."

The particulars in the second table "comprise the side affected—the part or parts of the lung diseased—the sex and the age of the patients—the form of the complaint, as far as it could be ascertained from the description—and the diseases with which some of the most remarkable morbid appearances were associated."

"*The Side*—

| | |
|---|---|
| The right was alone affected, in | 43 cases. |
| The left | 40 .. |
| Both sides were affected | 60 .. |
| The side was not mentioned | 2 .. |
| | 145 |

"*The Parts.*

| | |
|---|---|
| The upper lobe was alone diseased, in | 13 cases. |
| The centre was alone diseased, in | 7 .. |
| The posterior | 4 .. |
| The base or bases alone | 49 .. |
| The lung was universally or generally diseased in { right, 17; left . 14; both . 34 } | 65 .. |
| Different parts diseased, or parts not specified | 7 .. |
| | 145 |

"*Sex*—

| | |
|---|---|
| There were of children | 10 cases. |
| .. .. females | 43 .. |
| .. .. males | 92 .. |
| | 145 |

"*Age*—

| | |
|---|---|
| There were children | 10 cases. |
| The age was below 20, in | 16 .. |

| | | |
|---|---|---|
| The age was above 20, and below 30 in | | 32 cases. |
| .. .. | above 30, and below 40 | 23 .. |
| .. .. | above 40, and below 50 | 23 .. |
| .. .. | above 50 | 33 .. |
| .. .. | not mentioned | 8 .. |
| | | 145." |

"*Form*—

| | | |
|---|---|---|
| Recent, active, or acute | | 30 cases. |
| Old, passive, or chronic | | 63 .. |
| Lobular (among which were specified) | acute . . 5; chronic . 3; purulent . 3 | 28 .. |
| Purulent or suppurative, inclusive of two defined abscesses | | 13 .. |
| Gangrenous, inclusive of three defined abscesses | | 11 .. |
| | | 145" |

*Complications.*

Suppuration of the lung existed in combination with :—

"1. (Abscess) Stricture of the urethra.
2. Ossification of the pericardium.
3. (Abscess) Fever and suppuration of the neck around the thyroïd gland.
4. Fever, and hydrocephalus.
5. Fever, bronchitis, and pleuritis.
6. Fever, arachnitis, and phlebitis.
7. None mentioned.
8. Disease of the colon.
9. Fever and arachnitis.
10. (Abscess) Malignant disease, and pericarditis.
11. Fracture of the leg, followed by suppuration.
12. Malignant disease of the tongue.
13. Osteo-sarcoma, amputation, and phlebitis.

"Gangrene of the lung was found in combination with the following diseases:

1. Empyema, and epilepsy.
2. Peritonitis, from extra-uterine fœtation.
3. Suppuration of the scalp, and secondary syphilis.
4. Diabetes, and pleuritis.
5. Malignant disease of the œsophagus.
6. Empyema of the opposite side, and fractured ribs.
7. None mentioned.
8. Fever, and phthisis.
9. Fracture of the leg, and pleuritis.
10. Stricture of the œsophagus.
11. Diseased kidneys, and pleuritis."

Dr. Hughes concludes by the following important practical deduction :—"That while, according to my own researches, pneumonia is double in nineteen cases per cent., phthisis affects both lungs more or less in ninety cases per cent. :—that while pneumonia was *confined* to the base of one, or bases of both lungs, in sixty-two cases per cent., tubercles were *confined* to the base of the organ in only one case out of two hundred and fifty ;—and that while the upper part of the lung was principally, primarily, or solely affected with tubercular deposit in ninety-four cases out of every hundred, pneumonia was confined to the same part in only five cases in the hundred."

These facts bear directly and powerfully upon diagnosis.

### II. ON HÆMORRHAGE, OCCURRING AFTER DELIVERY, AND COMPLICATED WITH DISEASE OF THE SPLEEN AND KIDNEYS.

Mr. Lever first relates three cases of enlargement of the spleen, attended with hæmorrhage after delivery, and then draws the following general conclusions:—

"1st. That in females affected with enlargement or disease of the spleen the uterus is predisposed to dilate, and therefore admits of the effusion of blood into its cavity.

"2ndly. That the blood so collected, coagulates, and excites considerable constitutional irritation, as marked by the accession of rigors, fever, &c.

"3rdly. That the fever so produced in course of time, (varying in different cases,) assumes the intermittent type, especially when the patients have previously suffered from ague. And,

"4thly, That such intermittent fever is curable by the same remedies as are successful in the treatment of pure and uncomplicated ague."

Mr. Lever then relates two cases in which the morbus Brightii was accompanied with considerable hæmorrhage after delivery, and infers,

"1st. That labour occurring in patients affected with morbus Brightii is generally lingering.

"2ndly. That in such patients, although the fœtus and its secundines may be expelled by the natural uterine efforts, and the uterus may for a time appear to contract, yet that is very liable to become relaxed, and distended with blood.

"3rdly. That in patients so affected, peritonitis of a more or less acute character is prone to occur."

### III. NOTE ON THE MICROSCOPIC GLOBULES FOUND IN URINE. By GOLDING BIRD, M.D. &c.

Microscopical examination of the urine often detects minute globular bodies, which may be mistaken for particles of pus. To prevent the occurrence of such an error, Dr. Bird has favoured the profession with a brief account of these globules.

"1. *The true pus-particle,* roughly granular on its surface, and evidently compound in its structure, becoming reduced on the addition of ammonia, to a mucous mass, readily miscible with water; in which, however, the particles may be discovered, apparently shrivelled. On the addition of a drop of acetic acid to a drop of purulent urine, and examining the mixture with the microscope, the particles will be found to be disintegrated and partially dissolved, leaving numerous minute transparent circular bodies, which have been regarded as the nuclei of the original particles: these are free from granulations on their surface, and about one-fourth the size of a blood-disc, or $\frac{1}{8000}$ inch in diameter; the urine containing any appreciable quantity of pus *being albuminous, and becoming opaque on the application of heat.*"

"2. *The true mucous globule,* generally rather smaller than the pus-particle, being about $\frac{1}{2000}$ inch in diameter, appearing circular, with a smooth well-defined edge under a low magnifying power, but becoming distinctly granular on the surface with a power of 250 diameters; so closely resembling the pus-particle, that it is scarcely possible to distinguish them, except from the fact of the granulations on the surface being fewer and less distinct, not being readily miscible with water, and presenting merely a less shrivelled appearance on the addition of ammonia. These globules are often found cohering together, forming a kind of imperfect structure; and in this state they are met with in the mucous cloud which forms in all healthy urine by repose."

3. *The large organic globule.*—Of frequent occurrence, "varying in size from $\frac{1}{1000}$ to $\frac{3}{1000}$ inch in diameter, with great difficulty distinguished from the pus-globule: these seldom, if ever, constitute a deposit, but are found free and floating in the urine. They are generally scattered, and often few in number; frequently not more than a dozen being visible at one time in the field of the microscope. They are not, perhaps, so evidently compound in their character as the pus-globule; but on the addition of acetic acid, become broken up, and leave minute transparent single globules, like those left by pus when similarly treated. This variety is probably identical with what has been described as muco-pus."

4. *The small organic globule.*—"They are apparently perfectly simple in their structure, and absolutely spherical, so that they are seen to roll over each other on the slightest motion of the microscope: they are free from granulations on their surface; are seldom so large as a blood-disc, being generally about $\frac{1}{3000}$ inch in diameter. They mix with water with the utmost readiness, and undergo no change whatever; save, perhaps, becoming more transparent by boiling for seven minutes in strong acetic acid. These globules often form a glistening white deposit at the bottom of the capsule in which the urine has been allowed to cool after being gently heated; and in this state closely resemble, to the naked eye, the oxalate-of-lime deposit."

Of these globules, "the first," says Dr. Bird, appears to be "essentially connected with an albuminous state of the urine; as, in all cases hitherto examined, the pus-particle is never secreted in the animal economy without the constant accompaniment of a drop of serum in which it floats, just as the blood-disc does in liquor sanguinis. The diagnosis founded on the presence of the pus-particle in the urine will, of course, vary with its probable source. The second, or true *mucous globule,* is found in all urine which has been allowed to repose for a short time, so as to allow the cloud diffused through it to become deposited."

If the mucous membrane of the urinary passages is irritable, these globules increase in quantity. If, besides, there is diuresis, or a frequent desire to pass urine, the mucous globule is replaced by the third species.

The *large organic globule* is found in abundance in the urine of pregnant women, especially during the latter months, and when there is a frequent desire to empty the bladder: it exists, also, in every case of ardor urinæ that Dr. Bird has examined, though not necessarily connected with any evidence of irritable bladder. Dr. Bird relates some cases in point. These globules, too, are almost invariably present in the morbus Brightii; "and these, on account of the albuminous character of the urine, are distinguished with difficulty from the pus-particle: a comparison of them, however, with a specimen of the latter, will generally render the difference tolerably evident. In several cases of malignant disease of the womb this globule has been found abundantly in the urine."

*Of the small organic globule,* Dr. Bird has met with only two instances. In each its appearance was fugitive, and both were those of menstruating women. "These globules much resemble in appearance the minute amylaceous particles met with in vegetable juices: they are about the size, and resemble in transparency and spherical figure, the transparent nuclei obtained by treating the pus-particle, or large organic globule, with acetic acid. Is it improbable that such may be their true nature?"

The *milk globule* Dr. Bird has never seen in urine, unless it has been added by the patient. The *ferment globule* is a secondary thing, frequent in diabetic urine that has been passed for some days, never found in it when recent.

IV.—CASE OF FATAL PLEURITIS, APPARENTLY THE EFFECT OF THE PRESENCE IN THE RIGHT PLEURA OF A PIECE OF IVORY, CONSISTING OF FOUR ARTIFICIAL TEETH, WHICH HAD BEEN SWALLOWED THIRTEEN YEARS BEFORE. By W. G. CARPENTER.

Mr. H. was afflicted from childhood with asthmatic bronchitis; and it appears that several branches of his family have fallen victims to pleuritic, pulmonic, or tracheal affections. About eight years ago he became assistant to Mr. Watts, Chemist, in the Edgeware-road, with whom he subsequently lived. Last Winter, when Mr. Carpenter first knew him, he was never free from fever. The symptoms increased in the Spring, and on the 13th of April last, Mr. C. was called to him, as he had been attacked with pain in the side and chest, which had that evening become so acute as to render coughing, speaking, and breathing, almost impossible. The respiration was short and hurried: pulse 140, rather wiry: skin hot and dry: tongue furred at the base and margin, red in the centre: bowels confined: cough troublesome. On the right side the anterior and posterior part of the chest was dull on percussion, and the respiratory sound was not audible: there was no dulness on the left side, and the respiratory murmur was loud, and accompanied with mucous rattle.

Mr. Carpenter bled him to eight ounces, and gave calomel, antimony and colocynth. The blood was cupped and buffed, and the symptoms not being materially relieved, the venæsection was repeated, with calomel, antimony and Dover's powder every four hours, and salines with squill and digitalis. Cupping and blistering succeeded the bleeding. But the pyrexia continued, with profuse perspirations, and pain in the side; whilst the Report of the 16th states "there is still dulness on percussion, and absence of respiratory murmur; in fact, not the least sound, whether healthy or morbid, is heard in the side: there is a slight external fulness over the posterior part of the ninth rib, which is œdematous to the touch."

Dr. Bright now saw the patient, and gave it as his opinion that there was pleuritic inflammation, with, probably, effusion. What was done, is of little moment, as no improvement resulted, and death took place rather suddenly, on the 19th.

*Examination of the Body.*—"The lower part of the right side of the chest still appeared prominent, and its surface œdematous: the pectoral muscles on this side were undergoing decomposition, whilst those on the left were sound. As soon as I passed the scalpel into the right pleura, a gush of very offensive gas escaped. The pleural cavity on this side contained five pints of sero-purulent fluid. The lung was collapsed, and flatly pressed against the bodies of the vertebræ: the pleura which covered it, as well as that of the parietes, was thickly coated with coagulable lymph, which peeled off in layers. On the outer surface of the lung was an old fistulous opening, large enough to admit the tip of my little finger: when cut into, the lung appeared to contain a number of tubercles, some of which had suppurated. Some of the bronchial rings were ossified. The left lung was emphysematous; it contained a number of small miliary tubercles: the lesser bronchial tubes were filled with mucus: the pleura on this side appeared quite healthy. The heart and pericardium were also healthy. The liver was large, and easily gave way when rubbed between the fingers: its under surface very dark: the gall-bladder contained very little bile.

"After I had completed the examination, I was removing the remaining fluid and coagula of blood that had escaped from the pulmonary vessels to replace the lung, when I came to an irregular substance, which, when examined, turned out to be, to our great astonishment, a piece of ivory worked into four artificial front teeth, covered with a brownish crust, with a pointed piece of silver rivetted into the upper part of the teeth, which had evidently assisted in fixing them to the upper jaw; the base of the silver rivet surrounded with wadding. At each extremity there are two holes, which no doubt once contained the wire that fixed this mass of false teeth to the adjoining sound ones."

The circumstances of this curious case were elicited from the father of Mr. H. and from Dr. Kelk, of Scarborough.

The patient had swallowed the teeth thirteen years before, in a fit of coughing.

"From Dr. Kelk's statement, which was likewise corroborated by that of Mr. Eccles of Brompton, near Scarborough, at that time a fellow apprentice of Mr. H———, it does not appear that his sufferings were materially increased after the accident, or that he was unable to attend as usual to business. The morning after it happened, he mentioned the circumstance to Mr. Champley, his master, who advised him to take an aperient, supposing the teeth had passed into the stomach; it was thought that the teeth had passed away by the bowels, unnoticed;—and then the circumstance gradually became forgotten. He had been subject from a child to asthmatic bronchitis, which, perhaps, from the constant efforts at breathing, had dilated the air-passages, and, with the coating of mucus that no doubt existed, might have rendered the parts somewhat insensible."

Mr. Carpenter observes:—"I again examined the œsophagus; and we were satisfied that there was neither a recent wound nor a cicatrix to be found; and the only opening through which it could have escaped into the pleura of the right thoracic cavity, where I found it, must have been the fistulous one in the corresponding lung."

"The piece of ivory no doubt gradually made its way, by suppuration, through the lung: after its escape, the passage became obliterated, leaving merely the outer orifice, for I could not pass the probe through any distinct channel: this adhesion might have taken place after the escape of the air, when the lung became collapsed. It is a strange thing that no hæmorrhage ever took place; although there must have been some sharp points about the teeth, one of which is still remaining."

### V. Observations upon Inflammation of the Aqueous Membrane of the Eye. By Joseph R. Bedford.

After some introductory observations, Mr. Bedford observes:—

"Inflammation, then, of the aqueous membrane appears to be essentially marked by the appearance of a general or partial, more or less intense, opalescence, and which, as might be expected, may have its origin in the corneal, iridal, or capsular portions; when, in the former, the greatest facility for diagnosis is afforded. As each portion is differently circumstanced, it is necessary to speak of them separately. When the corneal is affected, it may be throughout, or in circumscribed patches, and most frequently in the inferior half;—and here the surgeon must be careful not to confound it with corneitis or corneal conjunctivitis. The points of diagnosis are these: An opalescence being observable, we must examine the cornea in different directions: a smoothness of its surface and clear reflection of light will at once exclude the conjunctival covering from any participation: a second inspection also, in different lights, will inform us that the opacity is deeply seated, and posterior to the corneal lamellæ; and if the disease be in its advanced stage, certain peculiar white spots, of which I shall speak more hereafter, will appear, and which may be considered as pathognomonic: although in corneitis—more especially, indeed, I believe I may say, only when chronic—the appearance is somewhat similar, consisting of a number of insulated opaque points, but which are less symmetrical, and more irregular in shape and disposition, than when a similar condition of the aqueous membrane is present, and the greater superficiality of which an experienced eye will soon detect. In noting, however, this deep-seated cloudiness, it must not be forgotten, that an opaque condition of the aqueous humour is incidental to advanced age, and that rare cases have been recorded by Clemens and Rosas of the same appearance being produced by a sudden suppression of the mammary secretion."

There can be little doubt that many cases of reputed iritis are merely inflammation of the aqueous membrane covering the iris. Mr. Bedford makes a good many observations on the point, which conclude, like the last chapter of Rasselas, without any thing being concluded. In fact it does seem to us that, for every thing like utility, it is just the difference between tweedledum and tweedledee, whether the iris, or a membrane so indissolubly united to it that both separation and demonstration are impossible, be *the* part affected.

The results of aquo-capsulitis appear to be,—1. *Increased secretion.* 2. *Effusion of lymph.* 3. *Puriform effusion.* 4. *Ulceration.* The disease seldom lasts long without producing one or other of these.

1. *Increased Secretion.*—This is the least frequent termination. There is a considerable increase of the distance between the cornea and iris; the latter assuming a concave appearance; the former appearing less convex, seeming, indeed, in its outline, to form a segment of the ocular, rather than one of a smaller circle. A tensive pain is produced, and some circum-orbital. There may, seemingly, be presbyopia *or* myopia. Mr. Bedford speaks thus of the treatment. He recommends "cupping to the amount of a few ounces; the administration of calomel, combined with rhubarb, antimony, or opium, according to the indications: blisters are also useful: colchicum has been strongly advised. Wardrop placed much value upon the evacuation of the aqueous humour; but this, although a palliative measure, has its disadvantages, and seems only absolutely necessary under two circumstances: 1. Where the distention of the cornea is so extreme as to threaten its health. 2. Where agonizing pain exists. But this evacuation, to be effectual, must be repeated daily; and even then is often useless. Rosas recommends that a small portion of cornea be actually removed. If the affection be of a strumous character, we must be regulated in our treatment by general principles, and prescribe, instead of the before-advised, alterative mercurials and tonics. It may be observed, that remedies useful in abdominal effusions are quite valueless here."

2. *Effusion of Lymph.*—This comprehends "all cases of morbid adhesion of the iris, either to the cornea or lenticular capsule: and this I believe to be the form with which we must connect the spotted or mottled appearance of the membrane; these spots appear actually to depend upon the presence of minute portions of lymph, either on its free or attached surface. They are distinctly visible, very circular, and uniformly diffused. Several authors, indeed, believe that these appearances owe their existence to an extension of inflammation to the proper substance of the cornea; but opposed to this is the fact, that they have been observed by Mr. Tyrrell on the iris: and, in a case related by Mackenzie, appeared and disappeared in the course of a few hours;—a circumstance difficult to account for, if their locality were in the cornea, a structure in which absorption is not rapid. In the least severe form the effusion exists in mere shreds, or as a fine false membrane."

3. *Puriform Effusion.*—Mr. Bedford *thinks* that pus *is* secreted by the aqueous membrane independently of ulceration.

"I have seen," he says, "one or two cases where, during the existence of hypopion, something very like a stream of pus was traceable from puriform points extending down to the hypopion. We do not in this form, at all events after the commencement, perceive the general opalescence before described, but simply a small number of isolated spots, more puriform in appearance, irregular in form, and less numerous, than those developed in the second form of the disease, and which have been described as conferring a mottled appearance. When they are once existent, I am not prepared to say whether they disappear in one part and appear in another; but I think, in the few cases which I have seen, they have, when once formed, been persistent until the termination of the disease. The spots may be more or less in number, possessing an intense opacity, which is gradually shaded off all round, until it subsides into the almost healthy clearness of the cornea. These appear to be only occasionally present.

"The color of the effusion, which is frequently a bright yellow,—its consistence, which is permanently fluid;—its continuing for some time freely moveable with every motion of the head,—its lying, moreover, for many days, I might almost say weeks, on the floor of the chamber, in contact both with the corneal and iridal membranes without any adhesion taking place, which would probably be the consequence of adhesive matter being so situated,—are strong proofs of purulent character. And I am satisfied that I have observed cases presenting these conditions, where the existence of the pus could neither be attributed to the suppuration of a lymph tubercle effused from the parenchyma of the iris, nor to the irruption into the anterior chamber of the contents of a corneal abscess; such effusion necessarily being the unassisted product of the membrane. Reasoning analogically, I need not observe, that, under certain circumstances, a serous membrane may pour out pus, or at least a very puriform fluid. The chief difficulty, however, seems to be the fact of its easy absorption. Now that these effusions, claiming the title of hypopion, and presenting the appearances I have described as characteristic of pus, are removed, there is no doubt; and if their removal be effected solely by the solvent properties of the aqueous humor, still the globules of pus must eventually enter the absorbent system."

4. *Ulceration.*—Of this Mr. Bedford does no more than announce the fact, and refer to Mr. Tyrrell for description. He winds up with the following summary:—

1. "Aquo-capsulitis may be simple or strumous—acute or chronic.

2. "Slight opalescence, or cloudiness of the membrane, is the essential mark of inflammation; and this is dependent upon no morbid deposition, but simply vascular turgescence, constituting inflammation, in a transparent structure.

3. "The peculiar spotted appearance of the membrane, depending probably on deposition of lymph, or insulated points of purulent effusion, together with turbidity of aqueous humor, is indicative of a more intense degree of inflammation.

4. "Aquo-capsulitis may terminate in increased secretion, fibrinous, and puriform effusion, and ulceration.

5. "Increased secretion, constituting dropsy of anterior chamber, is distinguishable by certain well-marked objective and subjective symptoms. Such a condition is not necessarily fatal to vision, and may, by certain modes of treatment, be relieved. Evacuation of aqueous humor, although a palliative measure, and deserving to be made use of when great suffering exists, is yet accompanied with many disadvantages, and, as far as a radical cure is concerned, is of very doubtful value.

6. "Fibrinous effusion is, in the first stage, amenable to simple remedies, and, when more advanced, yields to moderate mercurial action.

7. "Puriform effusion probably exists as an occasional result of simple inflammation, but less frequently so than fibrinous, that, when it exists to a great extent, evacuation may be resorted to with advantage, but that, in a great majority of cases, its removal is effected by absorption, and chiefly through the agency of those curative means which may be proper for the particular ophthalmia in which it occurs.

8. "And lastly, that, of all results, ulceration is the least frequent."

### VI.—Observations on the Diseases of the Orifice and Valves of the Aorta. By Norman Chevers, M.D.

Dr. Chevers informs us that he has "long been in the habit of examining the orifice and valves of the aorta, whether diseased or otherwise, in every case that has come under my notice; comparing the condition of these parts with concomitant states of other portions of the vascular system; and, as far as possible, remarking the relation between their appearances and the symptoms which were noticed during life." He has been led to form conclusions opposed to views at present generally received.

*Dilatation of the Aortic Orifice.*—Dr. Chevers points out the necessity of an acquaintance with the fact, that the normal dimensions of the aorta differ just above and just below the sigmoid valves. In eight healthy aortæ, "the mean circumference of the canal immediately below the valves was 36½ lines; that precisely above, 34 lines. But upon traction being made (after separating the vessel from the heart), the latter portion was found to be dilatable to 43 lines, the former only to 39½; proving, that while the lower part of the ostium is the wider, the upper is by far the more dilatable."

The lower portion of the orifice is almost entirely destitute of elasticity, and when the heart is flaccid can scarcely be stretched to more than three lines beyond its usual circumference by any sudden force less than sufficient to cause rupture of the part. Yet it is liable to great permanent enlargement in obstructed states of the arterial circulation, and, probably, the circumstance that the fibres here are chiefly parallel to the axis of the vessel prevents the opening regaining its proper dimensions.

The superior portion of the orifice is very elastic and dilatable. Yet it is very prone to permanent dilatation, and the dilatability of the vessel would seem to be generally lessened in nearly exact proportion to its increased diameter.

"*Dilatation of the lower portion of the orifice* becomes developed to a greater or less extent in nearly all cases of eccentric hypertrophy, or active aneurism of the left ventricle. It is also a very frequent accompaniment of simple dilatation of that cavity; but its occurrence in such cases is not invariable." "The most common immediate cause of dilatation of this part of the vessel is, either thickening and contraction of the aorta above or behind the valves, or disease of the sygmoïd crescents themselves interfering with the free emptying of the ventricle. In the latter case (providing the valves have not become so rigid as to prevent the impulse of the ventricle from being transmitted to the contents of the artery) there is usually observed a simply narrow condition of the upper part of the ostium and remainder of the aorta."

*Dilatation of the Superior Part of the Ostium.*—This "usually results from obstruction existing either in the main trunk, or in some of the terminal branches of the aorta. It is generally found in cases of thoracic and abdominal aneurysm; in stricture of the descending arch, where the ductus arteriosus is closed; in association with extensive ossification of the smaller arteries; and in the subjects of chronic, renal, and hepatic disease."

It is only when the vessels between the heart and the impediment have become stretched to the utmost that the ventricle begins to suffer dilatation. Previously to the occurrence of this, "there is dilatation of the superior part of the orifice, with a smaller state of the inferior, but not with actual contraction of this part: for it appears, that when the left ventricle first has the task of propelling the blood through obstructed arteries, its walls are apt to become greatly thickened, but its cavity retains, for a considerable time, nearly its original size." Aneurysmal conditions of the arteries of the limbs and of the abdominal aorta are usually attended with far more hypertrophy of the left ventricle, and with a smaller size of the inferior part of the ostium, than are similar states of the arch and descending thoracic trunk of the vessel.

*Equal widening of the Superior and Inferior Portions of the Orifice* mostly occurs only in a class of extreme cases, when the disease is verging towards a fatal termination. Then, "the cavity of the left ventricle is usually found very capacious, but its walls still remain considerably thickened: the aorta has become immensely and irregularly widened, and almost entirely inelastic, from the presence of extensive layers of bone and atheroma; proving that the arterial disease has been of very long standing. Coincident with this state, more or less contraction or retroversion of a portion, or the whole of the sygmoid curtains is usually observed—conditions which must, of course, have permitted reflux of blood through the widened orifice. But it is an interesting fact, that the whole of the orifice may become dilated to the utmost extent without the valves failing to perform their office, providing that, during the vicissitudes of the disease, their structures have not suffered any material lesion; for (the elastic tissue of which they are composed being capable, like most other parts of the vascular apparatus, of undergoing great extension under pressure gradually applied) they become widened and deepened in proportion as the dilatation of the aorta is increased."

When the valves are contracted and thickened and inefficient, the disease is much accelerated, the patient becoming liable to die with suffocative symptoms, from failure of the heart's action, or obstruction to the pulmonary circulation.

" Where the upper portion of the aortic ostium is more dilated than the lower, the most usual condition of the valves is, either great lengthening of their marginal cords, or depression of their superior points of attachment, with shallowing of the pouches, and rounding, or, occasionally, more or less retroversion of their free borders.

" Still, it is perfectly demonstrable, that so long as the inferior part of the orifice retains its proper dimensions, dilatation of its superior portion may go on to a very great extent without being attended by regurgitation through the valves, providing these curtains have not become retroverted, or suffered laceration. For I have observed, that, in such cases, a singular natural adaptation of the parts comes into play, to obviate this ill consequence: the upper margins of the sygmoïd curtains having been stretched laterally, their crescents, even though rendered rather shallower than natural, will still perform their valvular office; as, having lost their usual horizontal position, they bag down into the still undilated opening of the lower part of the ostium, and, their edges there coming into opposition, the vessel is perfectly closed against the reflux of blood into the ventricle."

He thinks *atrophy of the semilunar valves* exceedingly rare. "Under ordinary states," he remarks, "of the circulation, every portion of the valvular apparatus of the great artery is put in motion at each contraction of the left ventricle; and, whether the quantity of blood thrown into the artery be scanty or excessive, all parts of the curtains must be put more or less upon the stretch, as often as the elastic force of the aorta presses the fluid downwards into the sinuses of Morgagni: hence portions of the valves can scarcely have an opportunity of becoming wasted from inaction.—On the other hand, the common diseases of the endocardium and interior of the aorta, whether arising from undue pressure of the blood, friction, inflammation, or any other of the usual causes of lesion, are mostly attended with thickening of the parts from depositions of lymph between the laminæ of their fibrous structures; and, while this change may be followed by defective nourishment of the tissues—as marked by softening, friability and loss of elasticity, producing a tendency to ulceration or rupture—these cannot be fairly looked upon as the states to which the term 'atrophy' is conventionally applied.

"Circumscribed dilatations of the valves of the heart have been attributed to atrophy of their elastic tissues; but, although dependent upon a loss of cohesive and resilient power in the diseased structures, these changes are commonly preceded and accompanied by an opaque, thickened, and tumid state of the membranes, and never, so far as I have seen, by any degree of wasting, until portions of surface have begun to be removed by an ulcerative process. I have never met with a state of the aortic valves which appeared to me to be fairly attributable to simple atrophy of their structures."

The not unfrequent appearance of small perforations near the upper points of attachment both of the aortic and pulmonary valves, which has been attributed to atrophy, Dr. Chevers believes to be congenital, depending merely upon an arrest of development in the curtains during an early period of intra-uterine life. The cribriform state of the sigmoids may be observed in the hearts of very young children. He has noticed it three times in examining the aortæ of children about four years old, in the remaining portions of whose circulating systems no trace of lesion was discovered; and he once remarked it in one of the aortic valves of an infant who died at, or shortly after, birth.

*Vegetations.*

Dr. Chevers is of opinion that the minute semi-transparent warty vegetations so frequently met with on the endocardium and aorta, are not mere deposits of fibrin on inflamed or abraded parts of the surface. "The unvarying and symmetrical form which these little bodies always present, their complete blending in with the serous membrane, and the fact (which I lately happened to observe) of their assuming a deep-yellow hue in jaundice, seem to give strong evidence in favour of their being duly organized growths sprouting from the surface, and not mere adventitious deposits from the blood, of accidental formation. Indeed, they have always appeared to me to be intended to retard, in various ways, the progress of diseased actions in the parts upon which they appear. Thus they are commonly found existing, in the form of narrow fringes, upon the auricular edge of the mitral orifice, where this opening has undergone the state of disease commonly known as 'button-hole contraction,' and upon the most projecting portions of the aortic sygmoïds,—parts more exposed than any other to attrition and injury during the vicissitudes attending active disease of the left ventricle. They here seem to be intended to protect the entrance and outlet of the ventricle from attrition, and from the almost invariable consequence of this action (when unduly exercised upon any particular part of the lining of the vascular system), the deposition of clots:—for I think it will be uniformly observed, that these little bodies never receive any coagulum or other adherent deposit upon their surfaces; their minute elastic papillæ are sufficiently pliable to offer scarcely any impediment to the passage of the blood; but it is evident that massive coagula forming around the orifice of a contracted mitral valve would greatly increase the impediment and hasten the disease to a fatal termination: the same obtains with regard to the aortic valves, which, as we have already seen, are frequently liable to the formation of clots below their surfaces of contact. Lastly, it is probable that they prevent the occurrence of adhesion between opposed surfaces of the valves. In contraction of the aortic and mitral orifices, there is often a great tendency to adhesion between portions of the curtains of the former, and to coalition of the extremities of the slit-like aperture which the latter usually forms; but it is observable, that, closely as these vegetations are often placed to each other, they never adhere; each arises from its own foot-stalk, immediately in contact with, but perfectly distinct from, those around it; and the smooth-pointed apices of all appear to admit of being continually brought roughly into contact with each other, without displaying the least tendency to adhesive inflammation."

*Contraction of the Aortic Orifice.*—Dr. Chevers gives a good account of this morbid change. He observes that partial constriction may occur in three situations—below, above, or within the valvular curtains. All tend, sooner or later, to produce dilatation of the left ventricle.

"1. The part of the orifice immediately below the valves is probably less frequently the seat of marked organic lesions than any other portion of the aortic outlet: still, it is liable to become generally rigid and contracted from inflammatory change; and one portion of its compass—the upper part of the larger mitral curtain, which is attached to the bases of two of the aortic valves—is occasionally found coated with masses of fibrinous and other deposit: this layer of fibrous structure is also apt to become hardened, and rather contracted, after attacks of endocarditis, and in cases of disease affecting the left auriculo-ventricular orifice; in this way forming a cause of narrowing of the lower part of the aortic ostium, which I believe to be frequently overlooked."

"2. The portion of the artery immediately above the valves is occasionally found surrounded by a complete raised zone of tough, semicartilaginous deposit, in the subserous tissue: this may occur without being attended with the slightest apparent disease of the upper part of the vessel." If this deposit ossifies, it forms a permanent obstruction to the canal; if not, Dr. C. suspects that dilatation of the upper part of the orifice is gradually produced.

"3. Lastly, the valves themselves may either have become adherent at portions of their edges, or have suffered extreme thickening from the conversion of nearly the whole of their curtains into rigid masses of calcareous substance—conditions which, in their degrees, are both equally productive of impediment to the passage of the blood from the ventricle."

In such cases, Dr. C. has seen the aorta much dilated, with very thin walls—a condition due, he thinks, to stagnation and accumulation of the blood in it.

"The mouth of the aorta may be contracted from birth, either owing to its valves having become blended with each other, so as to present a mere funnel-shaped tube, with a slit at the apex through which the blood passes with difficulty; or from the whole of the vessel being merely below the ordinary size, as is sometimes the case where free communication has continued to exist between the pulmonary artery and the aorta. In no case has the aorta been found quite impervious nearer to the heart than immediately distal to the left subclavian artery: but partial stricture, and complete obliteration of the canal at this spot, or immediately below the ductus arteriosus, have occurred in many instances."

Dr. Chevers observes that "there are several classes of cases, occurring in patients of various ages between the commencement of puberty and the decline of life, in which, exclusively of any evidence of disease in its tissues, the whole of the aortic trunk, with its appendages, is found unusually small; often with coincident morbid narrowness of the left auriculo-ventricular orifice, lung-obstructions, and evidences of retardation of the blood in the right cavities of the heart, or in the veins and abdominal viscera."

The usual explanation has been that the small size of the aorta has led to these visceral congestions, by the plethora of the venous system. But Dr. Chevers believes with Dr. Barlow that the aortic diminutiveness has been the cause, not the consequence. Dr. C. states that this aortic narrowness may be occasioned by any condition of the thoracic viscera, or parts immediately covering the heart, which tends completely to embarrass or fetter the action of that organ, and he enumerates "malignant disease of the lungs, large collections of serum or pus in the pericardium or in the pleural cavities, various diseases of the anterior mediastinum, and excessive distention of the abdomen from ascites or hydatid disease, pushing up the diaphragm, and diminishing the cavity of the chest; but complete adhesion of the pericardium is, if not the most common, by far the most characteristic cause that could be cited."

Dr. Chevers alludes to the common opinion that adhesions of the pericardium gives rise to hypertrophy and dilatation of the heart. He disputes this, and that such enlargement occurs unless there has been also valvular disease. He confidently states that, during the last seven years, no case of decided hypertrophy with dilatation of the heart, occurring in combination with long-standing obliteration of the pericardial cavity, has been inspected at Guy's Hospital, in which there was not also present, either morbid narrowing of the aortic or mitral orifice, or some evident cause of obstruction to the pulmonary or systemic arteries—causes fully sufficient in themselves to produce enlargement of the heart's cavities in each case, quite independently of the adherent state of the pericardium.

2. *Diminution of the Aorta may result from any Obstinate Impediment to the Pulmonary Circulation.*

In chronic disease of the bronchial tubes Dr. C. has often found that, with more or less engorgement, dilatation, or thickening of the right cavities of the heart, there has been very perceptible diminution of the left ventricle and the aorta. Besides these, there are other cases "in which some permanent obstruction to the free passage of blood through the lungs having existed for many years, or perhaps almost from birth, in the form of thoracic or spinal malformation, or inadequate development of the pulmonary apparatus itself, the left cavities of the heart and the aorta have a tendency to remain considerably below the ordinary dimensions, from never having been called upon to perform their part in the circulation with vigour and freedom."

3. *Morbid Contraction of the Left Auriculo-ventricular Orifice has a tendency to be followed by Diminution in the Capacity of the Aorta and Left Ventricle.*

Dr. Chevers observes that there are three conditions which, with contraction of the left auriculo-ventricular orifice, the ventricle and aorta may assume.

2. "Where the patient's constitution is good, his muscular system firm, and the arterial circulation free, the cavity of the left ventricle becomes lessened in capacity in proportion to the degree of contraction in the mitral orifice, and the aorta undergoes remarkable diminution in calibre throughout the whole of its course. In these cases, the muscular tissue of the ventricle is generally firm and powerful; and the whole adaptation is evidently designed to enable the cavity to propel the blood in small quantities at a time, but with more than usual rapidity."

2. "But when, in such a case as the last, the obstruction behind the ventricle, or any other cause, has produced œdema, ascites, or congestive or organic visceral disease, the arterial circulation becomes impeded, and the aorta suffers dilatation; the cavity of the ventricle remaining small, until the artery has dilated to its utmost, and then the muscular walls of the cavity begin to yield."

3. "Where, as not unfrequently happens in these cases, the walls of the ventricle have previously become weakened, either from constitutional or local causes, the occurrence of any action tending to impede the arterial current is not succeeded, at first, by widening of the aorta, but the enfeebled ventricle undergoes dilatation passively, not appearing to have sufficient force at command to enlarge the artery. In such cases we occasionally find the left ventricle enormously dilated, without proportionate thickening of its walls, the whole of the orifice and canal of the aorta remaining narrow."

4.—*Any condition which greatly diminishes the quantity of the blood circulating through the system. Several states of this description might be noticed; but perhaps the two most characteristic, are, Malignant Disease, and Pulmonary Consumption.*

"I have usually remarked, that where death has occurred, either during the earlier stages of general malignant disease of the viscera, or where the fatal mischief has been confined to the extremities, or surface of the trunk; or, again, where it has been circumscribed in its extent, as in carcinoma of the mammary gland, stomach, uterus, or bowel, the heart and its appendages have been found small, with a dry shrivelled aspect, and an appearance of having shrunk from their ordinary dimensions."

The same fact appears to have been noticed by Louis.

"But this condition of the heart and its vessels is liable to become reversed in more general and advanced forms of malignant disease, and especially in cases of extensive hæmatoid deposits in the lungs or abdominal viscera, where softening has occurred. In such instances, I have found the cavities of one or both sides of the heart, with their arteries, the aorta more especially, dilated and thickened in various degrees; the latter vessel not unfrequently presenting traces of recent disease in its lining and subserous tissue. These changes appear to be traceable to the difficulty which the heart has experienced in propelling its contents through the vessels of the obstructed organs, owing to the diseased and often impervious condition of the capillaries which enter the softened masses."

Several authorities have taken notice of the diminution of the size of the heart and its vessels in phthisis. But usually the diminution of the left ventricle is the most remarkable. "In proportion to its narrowed cavity, the walls of the ventricle are often of remarkable thickness and strength; and though permitting the entrance of an extremely small quantity of blood at each diastole, its contractions are performed with a degree of activity and force which appears to prevent the aorta from becoming greatly diminished from inaction."

The Paper is a very valuable one.

### VII.—On the Operation for Cataract. By John Morgan, F.L.S.

Mr. Morgan introduces to our notice a modification of the operation for Depression of the Cataract, recommended to him by Mr. C. C. Egerton, Medical Superintendent of the Eye Infirmary at Calcutta. It would seem to be very successful, the cataract having little tendency to rise again. The operation is thus described:—

"The pupillary artery having been dilated by the application of belladonna to the eyebrow, the patient is to be placed as in cases of operation for depression, the eye to be operated upon being towards the light, and the head of the patient fixed by an assistant opposite to the breast of the operator, and against his own. I always prefer the sitting to the recumbent posture, except in operations for extraction, as there is an advantage gained by seeing down into the globe as you depress the lens to its destination. The needle which I use is extremely fine, of the same thickness from the point to the handle; cutting at the point, but not at the sides, and hardly longer than the diameter of the globe. In all operations on the eye, I invariably use a very short needle: which is, in my opinion, much more convenient than one of the usual length. The point of the instrument is now to be passed through the sclerotic, at the distance of rather more than a line from its junction with the cornea, and just below its transverse diameter: it is then to be carried, with a slight inclination forwards, directly through the central substance of the cataract, completely transfixing the lens. This part of the operation requires a good deal of careful management: the object will be to disturb surrounding parts as little as possible, by transfixing the lens *in situ;* and this is to be effected, not by pushing the point of the needle at once directly onwards, but by carefully drilling its way through the opaque body by rotating the handle of the instrument as it is held between the thumb and fore-finger, while the point at the same time is gently urged onwards. Having thus insured complete transfixion by drilling, the first step of the operation is accomplished; and it will be seen that the second can now be performed with the greatest ease and precision—I mean the dislocation and depression of the lens, which must of necessity follow the exact course of the point of the instrument by which it is impaled: and as the needle has been introduced below its transverse diameter, it will be *pulled*, instead of *pushed* down, and thus be effectually prevented turning on its axis into the vitreous humour as it is descending—an occurrence which will sometimes take place during the operation as at present performed, and which, for obvious reasons, it is desirable to guard against. Another advantage gained by transfixion is, that all chance of injuring the retina by the pressure of the lens, either from its slipping from the point of the instrument during the operation, or from its having been unconsciously left resting on that membrane after its completion, all danger of amaurosis from such causes, is removed; for we have such perfect command over the opaque body, that we can determine to the greatest nicety the exact course it will take in its descent, as well as the precise situation in which it will be left after the needle is withdrawn. The course should be so directed, that the anterior surface of the lens passes hardly a line distant from the corpus ciliare and retina, taking a curved sweep as it descends, corresponding with the concave curvature of the interior of the globe; and it should be left as nearly as possible with its upper circumference a line below the lower edge of the widely-dilated pupil. Very great care is required in disentangling the instrument from the cataract as it is being withdrawn from the globe; and this should be done by drilling the needle *out* from the depressed lens as it lies, without changing the direction of the handle of the instrument till it has been liberated in the same manner as it has been drilled *in* while the lens was *in situ;* for by neglecting this precaution, the lens will follow the point of the instrument, and be either raised again or forcibly dragged against the retina as the instrument is drilled out. Should any portion of an opaque capsule remain in the pupillary aperture after depression, it should be brought into the anterior chamber, if possible, with the point of the needle, before it is withdrawn from the globe, and after it has been disentangled from the lens. But I would recommend caution in cutting up a *posterior* capsule, lest the cells of the vitreous humour should be broken down in doing so: for, as I have already stated, I consider the preservation of their integrity very essential to the *success* of an operation for depression."

---

### ON THE RADICAL CURE OF VARICOCELE. By A. VIDAL, (DE CASSIS.)*

M. Vidal begins by stating that he has always been averse to operations on the veins. This aversion was inspired by a principle of operative medicine which is too often lost sight of, viz.—*No operation, attended with danger, ought ever to be performed, except for severe lesions incompatible with life.* For this reason, M. Vidal was in the habit of refusing to operate for varicocele; this, however, he no longer does, and for the following reasons.

1. M. Vidal's position as surgeon of the Hôpital du Midi has enabled him to see that varicocele is by no means a disease of old people, but that it frequently arises in early youth, and rarely begins after the age of thirty. This circumstance of the youth of the patients afflicted with varicocele, establishes a fundamental distinction between these kinds of varices, and those which accompany old age. From this pathological, ought to spring a therapeutic distinction. It would be right to run more risk in an operation to remove an infirmity in a youth who would otherwise have to support it during the course of a long life,

---

* Annales de la Chirurgie, Sept. 1842.

than in an old man, accustomed to his burden, and part of whose existence it may be said to form.

2. Varicocele is not merely a source of inconvenience, it may be extremely painful, and prevent the least exercise, and may thus render existence a burden to the rich man, and prevent the poor man from gaining a livelihood. Moreover, it is in youth especially that varicocele is attended with most suffering.

3. It is necessary to take into account also, the moral torture which is frequently, worse than the physical pain; thus youths of high rank in society will frequently entreat most vehemently to be relieved of the disease by extirpation. The sterility with which many men with varicocele are affected, is also a powerful motive for performing the operation.

4. A new argument in favour of the operation, and which distinguishes varicocele from other varices, is the absence of the varicose diathesis in the greater number of instances.

It is rare to meet with enlarged veins of the legs, with piles, or other varicose affections combined with varicocele. Observation tends to prove that varicocele in youth is a consequence of abuse of the genital organs, of masturbation, or other excessive excitations of the testicle. Thus the cause is local, and consequently the operation is much more applicable, and relapse less to be feared. In these cases not unfrequently the affection takes a highly acute form, thus a case is related, in which a youth of nineteen, who had indulged in excessive venery from the age of thirteen, had a very large varicocele developed rapidly after the occurrence of gonorrhœa with swelled testicle.

5. A still more powerful argument is the want of risk in the operation as now modified. M. Reynaud, of Toulon, practised this operation hundreds of times, without observing any serious accident. As a proof, M. Vidal adduces the following case. A youth had varicocele; the operation was performed; every thing goes off well. He then requests to be operated on for phymosis, and this operation, which is one of the most simple, produces phlebitis, and mortification of the skin of the scrotum. Thus a ligature of the veins succeeds, whilst a simple incision of the prepuce is attended with the most grave results.

The operation, as practised by M. Reynaud, was as follows. The operator seizes with both hands the diseased spermatic cord, seeks for and separates the vas deferens; then pinching up the scrotum with the fore-finger and thumb of the left hand, so as to embrace the spermatic vessels and nerves, he pierces the fold thus formed, at its base, with a curved needle armed with a waxed thread. The scrotum being let go, exhibits an interval of about an inch between the points of entrance and exit of the instrument; the two extremities of the thread are then brought together, and the ligature of the parts comprised in the loop which it forms, is conveniently tied upon a thick cylinder of linen, which is to be interposed between the knot and the skin. It is necessary to tie the ligature in such a way as to enable you easily to undo it, and loosen it in case the compression of the parts should be too great. Some simple dressing is to be applied to the punctures, and a light compress applied over the whole.

In a short time, some inflammation is developed in the parts touched by the thread and embraced by the ligature, but it is usually of short duration, and allows, in two or three days after the operation, of the ligature being tightened upon a new cylinder of lint, as the first is stained by the suppuration which begins to be established. If however the inflammation should extend, and the pain be great, the ligature must be loosened, and no fresh constriction employed until, by the application of emollient cataplasms, this state of inflammation has been subdued, which will certainly be the case in the course of two or three days.

In proportion as the soft parts are divided before the thread which presses on them, and as they cicatrise behind at the same time, the ligature is tightened occasionally, and this is not difficult, if care has been taken in tying the ligature in the first instance. From the fifteenth to the eighteenth day, the vessels and nerves of the testis, as well as the tunics which envelope them, are divided, and the skin alone remains; M. Reynaud then, in order to leave no doubt as to the complete division of the vessels of the cord, introduces a blunt-pointed bistouri, and divides the portion of skin which the ligature had left.

A simple wound remains, and this rapidly cicatrises, so that at the end of twenty-five days from the commencement of the operation, the cure is usually complete.

The modifications of the above-described operation which M. Vidal has adopted, consists in employing, instead of an ordinary thread, a silver wire, of about the thickness of a fine pin. This metallic thread allows of the ligature being tightened or loosened, without undoing the knot. After being fastened over the cylinder of linen as before, it can easily be twisted so as to allow of any degree of compression. Instead of a curved needle, M. Vidal employs a straight one, as being more convenient, and in place of cutting the skin which remains between the two punctures, he withdraws the thread as soon as the vessels are completely divided.

When there is any fear of a relapse, instead of a single thread, two may be passed, at a distance from each other of about two inches. The upper one alone is to be tied; the other, the one nearest the testicle, is left loose; this is the ligature "*d'attente.*" This ought to be kept in as long as possible, as, if, after the first ligature has divided the veins, when the patient begins to walk about, any tendency to a relapse is observed, the second thread may then be tightened, and a radical cure ensured.

The author adduces two cases in proof the success of this operation.

Not content, however, with employing the ligature in the above-mentioned cases, M. Vidal intends to employ this method of proceeding in cases of incurable tumors of the testicles and other parts similarly situated. Thus if the ligature does not produce a definitive cure of the tumor, it may check its further progress, and if it be a "*degenerescence,*" may prevent its propagation to other parts. In every case, it may be preparatory to an extirpation, giving a greater chance of success to this last operation. It will then be necessary to tie not merely the veins, but the whole cord, vas deferens, spermatic artery and nerves, *tout compris.* The success attending the ligatures which he has already attempted, makes M. Vidal presage as satisfactory a result for the ligature "*en masse.*"

With regard to this form of operation, however, the author owns that he has as yet had only two cases with any success, one already published, and another which will be published hereafter. When a person has only two facts on which to rest a new doctrine on the treatment of tumors, M. Vidal thinks it as well to speak with some reserve! (With this last observation we cordially agree.)

